# Biofilm formation is not associated with worse outcome in *Acinetobacter baumannii* bacteraemic pneumonia

**DOI:** 10.1038/s41598-018-25661-9

**Published:** 2018-05-08

**Authors:** Yung-Chih Wang, Tzu-Wen Huang, Ya-Sung Yang, Shu-Chen Kuo, Chung-Ting Chen, Chang-Pan Liu, Yuag-Meng Liu, Te-Li Chen, Feng-Yee Chang, Shih-Hsiung Wu, Chorng-Kuang How, Yi-Tzu Lee

**Affiliations:** 1 Division of Infectious Diseases and Tropical Medicine, Department of Internal Medicine, Tri-Service General Hospital, National Defense Medical Center, Taipei, Taiwan; 2 Institute of Clinical Medicine, School of Medicine, National Yang-Ming University, Taipei, Taiwan; 3 Department of Microbiology and Immunology, School of Medicine, College of Medicine, Taipei Medical University, Taipei, Taiwan; 4 Graduate Institute of Medical Sciences, College of Medicine, Taipei Medical University, Taipei, Taiwan; 5 National Institute of Infectious Diseases and Vaccinology, National Health Research Institute, Maoli County, Taiwan; 6 Department of Emergency Medicine, Taipei Veterans General Hospital, Taipei, Taiwan; 7 Division of Infectious Diseases, Department of Internal Medicine, Mackay Memorial Hospital, Taipei, Taiwan; 8 Department of Medical Research, Mackay Memorial Hospital, Taipei, Taiwan; 9 Division of Infectious Diseases, Department of Internal Medicine, Changhua Christian Hospital, Changhua, Taiwan; 10 Graduate Institute of Life Sciences, National Defense Medical Center, Taipei, Taiwan; 11 Division of Infectious Diseases, Department of Medicine, Taipei Veterans General Hospital, Taipei, Taiwan; 12 Institute of Biological Chemistry, Academia Sinica, Taipei, Taiwan; 13 Faculty of Medicine, School of Medicine, National Yang-Ming University, Taipei, Taiwan

## Abstract

The effect of biofilm formation on bacteraemic pneumonia caused by *A. baumannii* is unknown. We conducted a 4-year multi-center retrospective study to analyze 71 and 202 patients with *A. baumannii* bacteraemic pneumonia caused by biofilm-forming and non-biofilm-forming isolates, respectively. The clinical features and outcomes of patients were investigated. Biofilm formation was determined by a microtitre plate assay. The antimicrobial susceptibilities of biofilm-associated cells were assessed using the minimum biofilm eradication concentration (MBEC) assay. Whole-genome sequencing was conducted to identify biofilm-associated genes and their promoters. Quantitative reverse transcription polymerase chain reaction was performed to confirm the expression difference of biofilm-associated genes. There was no significant difference in the clinical characteristics or the outcomes between patients infected with biofilm-forming and non-biofilm-forming strains. Compared with non-biofilm-forming isolates, biofilm-forming isolates exhibited lower resistance to most antimicrobials tested, including imipenem, meropenem, ceftazidime, ciprofloxacin and gentamicin; however, the MBEC assay confirmed the increased antibiotic resistance of the biofilm-embedded bacteria. Biofilm-associated genes and their promoters were detected in most isolates, including the non-biofilm-forming strains. Biofilm-forming isolates showed higher levels of expression of the biofilm-associated genes than non-biofilm-forming isolates. The biofilm-forming ability of *A. baumannii* isolates might not be associated with worse outcomes in patients with bacteraemic pneumonia.

## Introduction

*Acinetobacter baumannii* is an important pathogen that causes nosocomial bloodstream infections and pneumonia, accounting for high morbidity and mortality rates^1,2^. *A. baumannii* is notorious for its multiple antimicrobials resistance. This multidrug resistance, along with its ability to form biofilms^3^, increases the difficulties in treating infections caused by this microorganism^4^.

Its prevalence in nosocomial outbreaks and device-related infections has been attributed to its ability to form biofilms in hospital environments and on medical devices attributes, respectively^4,5^. The ability to produce biofilms is a decisive advantage for colonization of different environments; thus, these bacteria can cause persistent infections^6^. In most institutions, the majority of *A. baumannii* isolates are isolated from the respiratory tracts of hospitalized patients^7^. A previous study reported that biofilm formation was observed in 95% of specimens obtained from patients who were mechanically ventilated for more than 24 hours, and *A. baumannii* and *Pseudomonas aeruginosa* were the most frequently isolated bacterial species^8^.

Biofilm-embedded cells are highly resistant to antimicrobials and disinfectants and therefore are difficult to eradicate^9–11^. However, whether biofilm-forming ability affects the clinical outcomes of *A. baumannii* pneumonia is largely unknown. This study aimed to investigate the correlation between the biofilm-forming ability of *A. baumannii* isolates and clinical outcomes of patients with *A. baumannii* bacteraemic pneumonia and to evaluate the microbiological features of biofilm-forming *A. baumannii* isolates.

## Results

### Clinical characteristics and outcomes of study population

We evaluated 856 patients from the 4 centers (CCH: 135, MMH: 132, TSGH: 186 and TVGH: 403) who had at least one episode of *A. calcoaceticus-baumannii* complex (Abc) monomicrobial bacteraemia during the 4-year study period. We excluded patients who had bloodstream infections caused by non-*baumannii Acinetobacter* spp., other concomitant infections at the time of presentation, or bacteraemia from a source other than pneumonia. Finally, 273 patients with documented *A. baumannii* monomicrobial bacteraemic pneumonia were included in the analysis.

**Figure 1:**
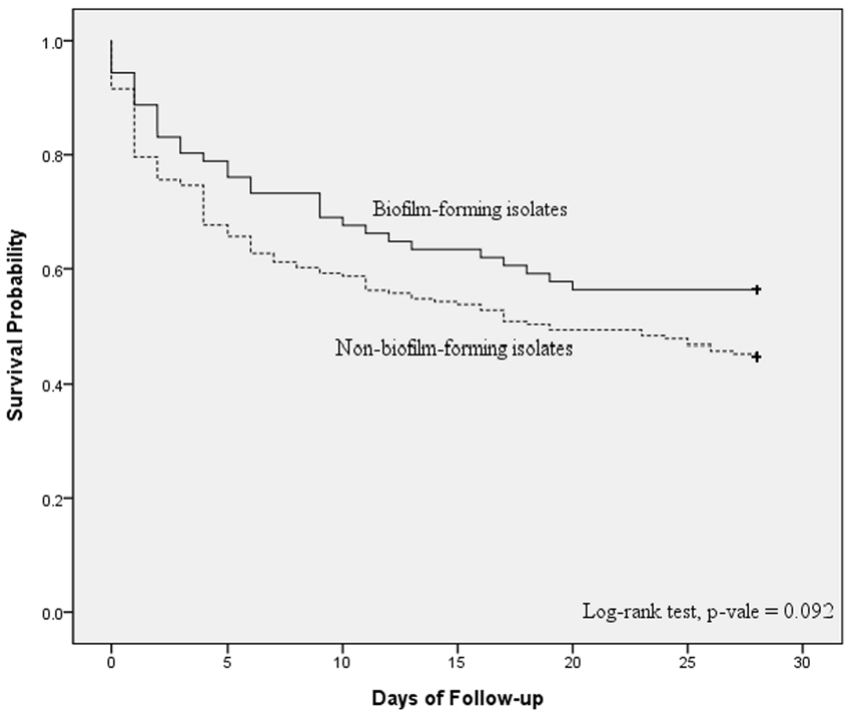
Comparison of Kaplan–Meier survival curves, at 28 days, between patients with *Acinetobacter baumannii* bacteraemic pneumonia caused by either biofilm-formation isolates or non-biofilm-formation isolates.

The demographic and clinical characteristics of patients with bacteraemic pneumonia caused by biofilm-forming (n = 71, 26.0%) and non-biofilm-forming (n = 202, 74.0%) isolates of *A. baumannii* are presented in Table 1. There were no significant differences in the demographic characteristics, comorbidities, invasive procedures, or previous antibiotic use between the two groups. The two groups had similar Charlson comorbidity index (CCI) score, disease severity (Acute Physiology and Chronic Health Evaluation II [APACHE II score]) and appropriateness of antimicrobial therapy. There was also no significant difference in the 14-day and 28-day mortality between the two groups by bivariate analysis (Table 1) or survival analysis (*p* = 0.092 by log-rank test; Fig. 1). Only two patients with non-biofilm-forming *A. baumannii* bacteraemic pneumonia had recurrent bacteraemia and no patients in the biofilm-forming group had recurrent bacteraemia.

**Table 1:** Demographic and clinical characteristics of patients with bacteraemic pneumonia caused by biofilm-forming and non-biofilm-forming *Acinetobacter baumannii*.

Characteristics	Biofilm-forming (n = 71)	Non-biofilm-forming (n = 202)	*p* value
Demographic characteristics			
Age, median (IQR), years	76 (59–82)	75 (59–82)	0.551
Male sex, no. (%)	55 (77.5)	151 (74.8)	0.648
Acquired in the ICU, no. (%)	32 (45.1)	105 (52.0)	0.317
Length of hospitalization before bacteraemia, median (IQR), days	22 (12–41)	19 (8.75–42)	0.572
APACHE II score, median (IQR)	25 (16–33)	26 (21–34)	0.326
Charlson comorbidity index, median (IQR)	3.75 (2–5)	3.67 (2–5)	0.817
Comorbid conditions, no. (%)			
Alcoholism	7 (9.9)	9 (4.5)	0.095
Cerebrovascular disease	13 (18.3)	33 (16.3)	0.702
Coronary artery disease	15 (21.1)	33 (16.3)	0.362
Congestive heart failure	11 (15.5)	36 (17.8)	0.655
Chronic obstructive pulmonary disease	15 (21.1)	44 (21.8)	0.908
Chronic kidney disease	22 (31.0)	70 (34.7)	0.574
Type 2 diabetes mellitus	26 (36.6)	66 (32.7)	0.545
Hypertension	36 (50.7)	85 (42.1)	0.208
Liver cirrhosis	8 (11.3)	15 (7.4)	0.316
Collagen vascular disease	6 (8.5)	8 (4.0)	0.140
Malignancy	19 (26.8)	57 (28.2)	0.814
Neutropenia	5 (7.0)	9 (4.5)	0.367
Immunosuppressive therapy	17 (23.9)	49 (24.3)	0.958
Recent surgery	13 (18.3)	54 (26.7)	0.156
Trauma	1 (1.4)	7 (3.5)	0.685
Septic shock	25 (35.2)	79 (39.1)	0.561
Invasive procedures, no. (%)			
Arterial catheterization	21 (29.6)	74 (36.6)	0.283
Central venous catheter	40 (56.3)	129 (63.9)	0.261
Hemodialysis	13 (18.3)	26 (12.9)	0.260
Nasogastric tube	21 (29.6)	84 (41.6)	0.089
Tracheostomy	12 (16.9)	29 (14.4)	0.606
Thoracic drain	5 (7.0)	14 (6.9)	0.975
Abdominal drainage	3 (4.2)	14 (6.9)	0.599
Mechanical ventilation	33 (46.5)	77 (38.1)	0.217
Previous ICU admission, no. (%)	44 (62.0)	145 (71.8)	0.123
Previous antibiotic exposure, no. (%)			
All	51 (71.8)	151 (74.8)	0.629
All beta-lactam	44 (62.0)	128 (63.4)	0.834
Aminoglycoside	10 (14.1)	25 (12.4)	0.711
Penicillin	7 (9.9)	12 (5.9)	0.264
Beta-lactam/beta-lactamase inhibitor	9 (12.7)	38 (18.8)	0.239
Cephalosporin	15 (21.1)	40 (19.8)	0.811
Anti-pseudomonas cephalosporin	23 (32.4)	50 (24.8)	0.211
Anti-pseudomonas carbapenem	14 (19.7)	53 (26.2)	0.272
Sulbactam	1 (1.4)	15 (7.4)	0.078
Fluoroquinolone	16 (22.5)	47 (23.3)	0.900
Tigecycline	8 (11.3)	15 (7.4)	0.316
Colistin	5 (7.0)	8 (4.0)	0.333
Macrolide	5 (7.0)	9 (4.5)	0.367
Clindamycin	1 (1.4)	13 (6.4)	0.124
Vancomycin	6 (8.5)	21 (10.4)	0.637
Teicoplanin	20 (28.2)	51 (25.2)	0.629
Appropriate antimicrobial therapy, no. (%)	19 (26.8)	57 (28.2)	0.814
Outcome			
14-day mortality	26 (36.6)	93 (46.0)	0.169
28-day mortality	31 (43.7)	112 (55.4)	0.087
Recurrent bacteraemia	0 (0.0)	2 (1.0)	1.000

^*^APACHE II = Acute Physiology and Chronic Health Evaluation II, ICU = intensive care unit, IQR = interquartile range.

Multivariate logistic regression analysis was performed for the overall cohort to delineate the independent risk factors for 28-day mortality in patients with *A. baumannii* bacteraemic pneumonia (Table 2). A higher APACHE II score and previous intensive care unit (ICU) admission were independently associated with 28-day mortality. Notably, no significant correlation was observed between biofilm formation capability and 28-day mortality (odds ratio [OR], 0.623; 95% confidence interval [CI], 0.361–1.074; *p* = 0.088).

**Table 2:** Logistic regression analysis of predictors for 28-day mortality among patients with bacteraemic pneumonia caused by *Acinetobacter baumannii*.

Characteristic	Univariate analysis		Multivariate analysis	
	Crude OR (95% CI)	*p*	Adjusted OR (95% CI)	*p*
Biofilm formation strains infection	0.623 (0.361–1.074)	0.088		
APACHE II score	1.152 (1.109–1195)	<0.001	1.158 (1.113–1.206)	<0.001
Previous ICU admission	2.022 (1.191–3.436)	0.009	2.723 (1.361–5.450)	0.005
Hypertension	0.606 (0.375–0.981)	0.041	0.437 (0.236–0.811)	0.009
Congestive heart failure	0.450 (0.235–0.862)	0.016		
Cerebrovascular disease	0.470 (0.244–0.903)	0.023		
Previous antibiotic exposure	1.695 (0.975–2.947)	0.061		
Multidrug re sistance strains infection^a^	1.684 (0.751–3.777)	0.206		

*APACHE II = Acute Physiology and Chronic Health Evaluation II, ICU = intensive care unit; CI = confidence interval, OR = odds ratio.

^a^Definition: resistance to three or more of the following classes of antimicrobial agents: anti-pseudomonal cephalosporins, anti-pseudomonal carbapenems, ampicillin/sulbactam, fluoroquinolones, and aminoglycosides.

As there is no universal standard reference value used for evaluating biofilm formation capacity, strains with optical density of 570 nm (OD_570_) values at least twice those of the negative controls were classified as biofilm-formation isolates in this study. In order to validate whether there is any bias in assessing the biofilm forming capacity, we conducted the following analysis. Initially, the results were divided into four following categories according to their optic densities as (1) strong biofilm producer (OD_570_ values ≧ 8 folds those of the negative controls); (2) medium biofilm producer (OD_570_ values 4–8 folds those of the negative controls); (3) weak biofilm producer (OD_570_ values 2–4 folds those of the negative controls); and (4) non-biofilm producer (OD_570_ values ≤2 folds those of the negative controls). We found that there was no significant difference in 14-day and 28-day mortality between the strong biofilm producer, medium biofilm producer, weak biofilm producer and non-biofilm producer (data not shown).

Then we tried to set the cut-off value higher. Specifically, those with the OD_570_ values at least 4-folds of the negative controls were classified as positive biofilm-formation isolates. Further analysis showed no significant difference in 14-day and 28-day mortality between the biofilm-formation and non-biofilm formation isolates (data not shown).

Next, we used the *A. baumannii* ATCC 19606 as a reference strain^12,13^. Strains with OD_570_ values greater than that of *A. baumannii* ATCC 19606 were considered positive for biofilm formation. Of the 273 isolates, there were 181 and 92 isolates with biofilm-forming capacity higher than *A. baumannii* ATCC 19606 and lower than *A. baumannii* ATCC 19606, respectively. The further analysis showed there was no difference in 14-day and 28-day mortality between the two groups (data not shown).

In the end, we conducted the air-liquid interfaces biofilm formation method to evaluate the ability of biofilm formation^3^. There were 66 isolates produced air-liquid interfaces biofilm while the others did not. We compared the 14-day and 28-day mortality between the air-liquid interfaces biofilm-formation and non-biofilm formation isolates. The results disclosed there was no significant difference in 14-day and 28-day mortality between the air-liquid interfaces biofilm-formation and non-biofilm formation isolates (data not shown).

Antimicrobial treatment and the outcomes of patients with biofilm-forming *A. baumannii* bacteraemic pneumonia are listed in Table 3. Patients who were treated with ampicillin/sulbactam or sulbactam had a lower 28-day mortality rate (33.3%) even though they had a higher APACHE II score than those who received other therapeutic regimens. Multivariate analysis revealed that no specific therapeutic regimen was independently associated with higher or lower 28-day morality rates. There was no identifiable link between the antimicrobial treatment regimens and the clinical outcomes in those with non-biofilm-forming *A. baumannii* bacteraemic pneumonia (see Supplementary Table S1).

**Table 3:** Antimicrobials usage and the 28-day survival rates in patients with bacteraemic pneumonia caused by biofilm-forming *Acinetobacter baumannii*.

Main agents used	Patients, no.	APACHE II score	Patients, no. (%)			28-Day survivors
		Median (interquartile range)	Appropriate antimicrobial therapy	Combination antimicrobial therapy	28-Day non-survivors	
Anti-pseudomonal penicillins^a^	10	22 (16.53–0.75)	5 (50.0)	1 (10.0)	7 (70.0)	3 (30.0)
Anti-pseudomonal cephalosporins^b^	16	26 (17.25–30.25)	4 (25.0)	1 (7.1)	6 (37.5)	10 (62.5)
Anti-pseudomonal fluoroquinolones^c^	3	39 (20–39)	0 (0)	0 (0)	3 (100.0)	0 (0)
Anti-pseudomonal carbapenems^d^	12	29 (21.75–35.75)	6 (50.0)	1 (8.3)	5 (41.7)	7 (58.3)
Ampicillin/sulbactam or sulbactam	6	36.5 (21.75–44)	1 (16.7)	3 (50.0)	2 (33.3)	4 (66.7)
Non-antipseudomonal β-lactamases^e^	11	19 (13–33)	0 (0)	2 (18.2)	4 (36.4)	7 (63.6)

^*^Data are the median (interquartile range) for continuous variables and number of cases (%) for categorical variables. APACHE II = Acute Physiologic and Chronic Health Evaluation II.

^a^Piperacillin, piperacillin/tazobactam and ticarcillin/clavulanate.

^b^Cefoperazone, ceftazidime, cefepime and cefpirome.

^c^Ciprofloxacin and levofloxacin.

^d^Imipenem and meropenem.

^e^Penicillin, amoxicillin/clavulanate, cefazolin, cefuroxime, cefotaxime, cefmetazole and flomoxef.

### Clonality and antibiograms of the *A. baumannii* isolates

To determine the molecular epidemiology of the causative pathogens, 64 biofilm-forming and 126 non-biofilm-forming *A. baumannii* isolates were randomly selected for pulsed-field gel electrophoresis (PFGE) analysis. There were 68 pulsotypes (P1–P68), based on a threshold of 80% similarity (see Supplementary Fig S1). The biofilm-forming and non-biofilm-forming isolates belonged to 37 and 44 pulsotypes, respectively and 28 biofilm-forming isolates shared the same pulsotype with non-biofilm-forming isolates. P5 (n = 36) and P4 (n = 23) were the most common pulsotypes.

The antimicrobial susceptibilities and the minimum inhibitory concentrations (MICs) of biofilm-forming and non-biofilm-forming *A. baumannii* isolates against the antimicrobials are presented in Table 4. The proportion of multidrug resistance (MDR) isolates was significantly lower in the biofilm-forming group and biofilm-forming isolates were more susceptible to most commonly used antibiotics, except ampicillin/sulbactam, piperacillin/tazobactam and tigecycline. The MIC_90_ of ceftazidime, cefepime or cefpirome, piperacillin/tazobactam, ampicillin/sulbactam, ciprofloxacin and meropenem were also lower in the biofilm-forming group. Ten biofilm-forming isolates that belonged to different pulsotypes were selected for the minimum biofilm eradication concentration (MBEC) assay (see Supplementary Table S2). The MBECs of imipenem and meropenem were much higher than the MICs of imipenem and meropenem in biofilm-forming isolates.

**Table 4:** Comparison of the antimicrobial susceptibilities of biofilm-forming and non-biofilm-forming *Acinetobacter baumannii* isolates.

Antimicrobial agent	Biofilm-forming isolates (n = 71)		Non-biofilm-forming isolates (n = 202)		*p value* ^b^
	Resistance, no. (%)	MIC_90_ (µg/mL)	Resistance, no. (%)	MIC_90_ (µg/mL)	
Amikacin	44 (62.0)	>2048	151 (74.8)	>2048	0.040
Gentamicin	57 (80.0)	>2048	181 (89.6)	>2048	0.043
Ceftazidime	56 (78.9)	512	186 (92.1)	1024	0.003
Cefepime or cefpirome	48 (67.6)	128	157 (77.7)	256	0.009
Piperacillin/tazobactam	52 (73.2)	4096/4	163 (80.7)	>4096/4	0.187
Ampicillin/sulbactam	46 (64.8)	64/32	141 (69.8)	128/64	0.434
Ciprofloxacin	55 (77.5)	256	187 (92.6)	512	0.001
Imipenem	39 (54.9)	64	139 (78.1)	64	0.035
Meropenem	39 (54.9)	64	142 (78.5)	128	0.018
Tigecycline	19 (26.8)	4	39 (19.3)	4	0.187
Colistin	0	2	0	2	—
Multidrug resistance^a^	58 (81.7)	—	188 (93.1)	—	0.006

^a^Definition: resistance to three or more of the following classes of antimicrobial agents: anti-pseudomonal cephalosporins, anti-pseudomonal carbapenems, ampicillin/sulbactam, fluoroquinolones and aminoglycosides.

^b^*p* value: The difference in resistance rate to antibiotics between biofilm-forming isolates and non-biofilm-forming isolates.

### The correlation between carbapenemase production and biofilm formation

Carbapenem-resistant *A. baumannii* strains were less likely to produce biofilms than carbapenem susceptible strains (17.0% *vs*. 38.6%, *p* < 0.001). To delineate the impact of carbapenem resistance on biofilm formation capability, we evaluated the biofilm formation capability of *A. baumannii* transformants harboring different carbapenemase genes. All the transformants had MICs of imipenem and meropenem ≥8 mg/L (data not shown). Carbapenem-resistant transformants containing vectors with various carbapenemase genes produced less biofilms than the parent strain *A. baumannii* reference strain ATCC 15151 (Ab15151) carrying empty vectors, except those harboring *bla*_OXA-58_ (see Supplementary Fig S2). To elucidate if decreased biofilm forming capacity also occurred with the expression of non-carbapenemase genes, a transformant containing a vector with *Acinetobacter*-derived cephalosporinase (ADC) gene was also generated and showed no significant decrease in biofilm formation capability compared with its parent strain Ab15151 carrying empty vectors (data not shown).

### Biofilm-associated genes of the selected isolates and quantitative reverse transcription polymerase chain reaction (qRT-PCR) assay

To investigate the correlation between biofilm-forming capacity and the presence of biofilm-associated genes, 9 biofilm-forming and 30 non-biofilm-forming clonally unrelated isolates were selected for whole genome sequencing. All biofilm-forming strains contained the known biofilm-formation-associated genes except two strains (isolate 156 and 160), which lacked the genes encoding *Bap1* and *Bap2* (see Supplementary Table S3). Interestingly, most of the non-biofilm-forming isolates also had biofilm-formation-associated genes (see Supplementary Table S3). The promoter sequences of biofilm-associated genes were not different between biofilm-forming and non-biofilm-forming strains (data not shown).

To determine whether the expression of biofilm-associated genes is different between biofilm-forming and non-biofilm-forming strains, we matched two biofilm-forming isolates with two non-biofilm-forming isolates that belonged to the same sequence type (ST) (ST 218 and ST 436). The qRT-PCR assay revealed higher expression levels of the biofilm-formation-associated genes (*Bap1*, *AbaI*, *Cus A/B*) in the biofilm-forming isolates compared with the matched non-biofilm-forming isolates (see Supplementary Fig S3). However, the expression levels of *Bap2* and *BfmS* were not different between biofilm-forming and non-biofilm-forming strains (data not shown).

## Discussion

The present study demonstrated no significant differences in the clinical characteristics and 14-day and 28-day mortality between patients with *A. baumannii* bacteraemic pneumonia caused by biofilm-forming and non-biofilm-forming isolates. Biofilm formation was not independently associated with higher or lower mortality. The biofilm-forming isolates were significantly more susceptible to most antimicrobials tested, including imipenem, meropenem, ceftazidime, ciprofloxacin and gentamicin. No specific regimen exhibited a significant 28-day survival benefit for patients infected with biofilm-forming isolates. Although both biofilm-forming and non-biofilm-forming isolates had biofilm-formation-associated genes and their promoter sequences, the biofilm-forming isolates expressed some of the biofilm-formation-associated genes at a higher level than non-biofilm-forming isolates.

Studies on the relationship between biofilm-formation phenotype and clinical infection outcomes are limited. This is the first study investigating the relationships between biofilm-forming capability and clinical outcomes of *A. baumannii* bacteraemic pneumonia. A previous study investigated 89 isolates obtained from various infection foci and found no significant difference on the mortality rate between those infected with biofilm-forming and non-biofilm-forming *A. baumannii*^14^. The heterogeneity of the infectious diseases and relative low number of cases provided weak evidence to demonstrate correlation between biofilm formation and clinical implication from that study. Another study investigated 221 clinical isolates and assessed the association between biofilm-formation ability and the clinical outcomes^15^. However, there were only 53 Abc isolates obtained in that study. Recently, Zhang *et al*.^15^ studied *A. baumannii* isolates from 121 patients with hospital-acquired pneumonia and concluded that the previous stay in the ICU, use of antibiotics and less severe disease were likely to lead to infection from biofilm-forming isolates. Although the number of cases was relative large, they did not mention the correlation between biofilm-formation ability and the clinical outcome. Besides, the *A. baumannii* isolates were obtained from sputum samples in their study. This could not exclude the possibility that these pathogens may just have been the colonizers. Since the clinical conditions and sample sizes were diverse among the above studies, they could not draw robust conclusions on the correlation between biofilm formation ability and the clinical implication.

In our study, all isolates were obtained from the blood samples, indicating true causative pathogens. Patients infected with biofilm-forming and non-biofilm-forming isolates had similar clinical characteristic and outcomes. The finding that biofilm-forming and non-biofilm-forming *A. baumannii* isolates shared the same pulsotypes ruled out the possibility that biofilm-forming isolates belonged to certain low-virulence pulsotypes. Further investigations are required to determine if biofilm-forming isolates are less virulent than non-biofilm-forming isolates belonging to the same pulsotype.

The biofilm-forming isolates in this study were significantly more susceptible to amikacin, gentamicin, ceftazidime, cefepime or cefpirome, ciprofloxacin, imipenem and meropenem. The results suggested a possible inverse association between antibiotic resistance and biofilm-forming ability. Previous reports showed that biofilm-forming *A. baumannii* were more frequently susceptible to imipenem and ciprofloxacin^13,14^. One recent study also demonstrated that strong biofilm-forming *A. baumannii* isolates exhibited low-level resistance to gentamicin, minocycline and ceftazidime^16^. Our results are consistent with these previous findings. While several attributes can confer antimicrobial resistance to bacteria within a biofilm, even less-resistant biofilm-forming bacteria can survive^6,17^. Based on the aforementioned results, it is likely that the biofilm-forming strains rely less frequently on intrinsic antimicrobial resistance for survival.

The biofilm-forming isolates in our study exhibited lower rates of carbapenem resistance than non-biofilm-forming isolates. Since the correlation between biofilm-forming capacity and carbapenem resistance in previous studies was questionable^18,19^, we compared the biofilm-forming ability of *A. baumannii* transformants harboring various carbapenemase genes to that of the parent strain with an empty vector to clarify this correlation. We found that most carbapenem-resistant transformants had decreased biofilm-forming capacity. Nevertheless, we could not find a similar trend in transformants carrying a vector with non-carbapenemase gene (ADC-type β-lactamase). The energy necessary for expressing the carbapenemase genes may decrease the biofilm formation, but not in the case of ADC genes. Research efforts should be directed to obtain a deeper understanding of this issue. Among *A. baumannii* expressed different carbapenemase genes, those harvesting *bla*_*OXA-58*_ produce more biofilm than other carbapenem-resistant transformants. It seems that the influence of carbapenemase genes on biofilm formation varied between *A. baumannii* expressed different carbapenemase genes. Further experiments are needed to elucidate its mechanism.

According to the results of the MBEC assay, the biofilm-forming isolates showed extremely high antibiotic resistance within the biofilm. Although biofilm-forming isolates were more susceptible than non-biofilm-forming isolates, the increased resistance of bacteria embedded in the biofilm might result in treatment failure and recurrent infection in patients infected with biofilm-forming isolates. Because of the high drug resistance of bacteria within biofilms, clinicians should manage infections caused by biofilm-forming isolates very carefully. Currently, there are no standard therapeutic guidelines for the management of *A. baumannii* biofilm-associated infections. Our previous *in vitro* study demonstrated that meropenem plus sulbactam showed synergism against biofilm-embedded carbapenem-resistant *A. baumannii*^20^. We analyzed therapeutic regimens and outcomes and found that patients who received ampicillin/sulbactam or sulbactam treatment had a more favorable 28-day survival rate in this study. However, we could not draw any conclusion due to the small number of cases. Additional large-scale clinical studies are needed to determine the optimal therapeutic regimens for *A. baumannii* biofilm-associated infections.

The whole genome sequencing analysis demonstrated the presence of biofilm-formation-associated genes and their promoter sequences in biofilm-forming isolates as well as non-biofilm-forming isolates. Further qRT-PCR experiments showed that the biofilm-forming isolates expressed higher levels of some biofilm-formation-associated genes. There may be several explanations for these findings. First, since the development of biofilms is regulated by a variety of factors, such as the environment, nutrition, stress and the pathogen itself, pathogens that contain biofilm-associated genes may only produce biofilms under certain conditions^21,22^. Second, some biofilm-associated proteins function in processes other than biofilm formation^5,23^. The presence of biofilm-associated genes in non-biofilm-forming isolates might indicate involvement in other physiological processes rather than biofilm formation. For example, *pgaABCD* encodes genes involved in the synthesis of poly-β-(1–6)-N-acetylglucosamine, which is an essential component of bacterial cell walls^24^. Third, the expression of biofilm-associated genes may be upregulated by factors other than promoters, such as transcriptional activators.

The limitations of this study included several confounding factors, such as variations in patient care and different patient backgrounds, which are inherent to a retrospective study. In addition, the biofilms established *in vitro* may be quite different from those in human bodies. It should be of concern that the results of our study may not be applied to the clinical settings. Yet, there is no standard laboratory protocol to assess *in vivo* biofilm formation. To overcome the inadequacy, we conducted a crystal violet assay, MBEC assay and genetic analysis to validate the biofilm-forming phenotype. We also conducted the qRT-PCR to confirm the expression of biofilm-associated genes. The inclusion of a large number of patients from multiple medical centers located in representative regions of Taiwan is another major strength of this study.

In conclusion, there might be no significant difference in the clinical characteristics and infectious outcomes between patients with bacteraemic pneumonia caused by biofilm-forming and non-biofilm-forming *A. baumannii* isolates. Although the biofilm-forming isolates were more susceptible to most antibiotics than non-biofilm-forming isolates, the biofilm-embedded cells indeed exhibited much higher antibiotic resistance. The antibiotic susceptibilities of biofilm-forming *A. baumannii* isolates should be interpreted cautiously. Further studies are required to determine the optimal treatment for bacteraemic pneumonia caused by biofilm-forming *A. baumannii*.

## Methods

### Study population

This retrospective study was performed during a 4-year period from January 2012 to December 2015 at four medical centers in Taiwan: Changhua Christian Hospital (CCH, 1676 beds) in Central Taiwan and Mackay Memorial Hospital (MMH, 2055 beds), Tri-Service General Hospital (TSGH, 1712 beds) and Taipei Veterans General Hospital (TVGH, 2900 beds) in Northern Taiwan. Patients who had at least one positive blood culture for *A. baumannii*, accompanied with the signs and symptoms of infection, were enrolled. Only the first blood culture from patients with two or more positive blood cultures was included. Patients under 20 years of age and those with incomplete medical records were excluded. The study protocol was approved by the institutional review boards (IRBs) of each site (CCH: IRB No. 140514, MMH: IRB No. 14MMHIS125, TSGH: IRB No. 1-103-05-100, TVGH: IRB No. 2015-04-001AC). All methods were performed in accordance with the relevant guidelines and regulations. Written informed consent was waived by the IRBs due to the retrospective nature of the analysis using information contained in medical charts and records, which were anonymized and de-identified prior to analysis.

### Data collection

Medical records were reviewed to extract patient information, including demographic characteristics, comorbidities, admission to ICU, duration of hospitalization and the schedule and doses of antimicrobials administered. The presence of arterial cauterization, a central venous catheter, nasogastric tube, tracheostomy, ventilator, thoracic drain, or abdominal drain for more than 48 hours prior to bacteraemia onset was also recorded. The inclusion criteria^25^ for *A. baumannii* bacteraemic pneumonia consisted of: (a) at least one positive respiratory sample (sputum, endotracheal aspirate, or bronchoalveolar lavage) for *A. baumannii* obtained within 48 hours before or after the first positive blood culture; (b) a clinical course compatible with the diagnosis of pneumonia as defined by Centers for Disease Control and Prevention guidelines^26^ and (c) the positive blood culture could not be attributed to another source of infection. The onset of bacteraemia was defined as the day when then blood culture that yielded *A. baumannii* was obtained. Episodes of bloodstream infection were considered acquired in the ICU if they appeared 48 hours after ICU admission. Chronic kidney disease was defined as an estimated glomerular filtration rate <60 mL/min/1.73 m^2^. Neutropenia was defined as an absolute neutrophil count <0.5 × 10^9^ cells/L. Immunosuppressive therapy was defined as use of immunosuppressive agents within 2 weeks or use of corticosteroids (≥15 mg of prednisolone daily for 1 week) within 4 weeks before bacteraemia onset. Recent surgery was defined as surgery within 4 weeks before bacteraemia onset. CCI was also calculated for further analysis. The severity of illness was evaluated using the APACHE II score within 24 h before bacteraemia onset. Previous ICU admission was defined as admission to ICU within 30 days before bacteraemia onset. Previous use of antimicrobials was defined as the use of antimicrobials within 30 days before bacteraemia onset. Appropriate antimicrobial therapy was defined as administration of at least one antimicrobial agent to which the causative pathogen was susceptible within 24 h after bacteraemia onset, via the approved route and at the recommended dosage for the affected organ(s). Antimicrobial therapy that did not meet this definition was considered inappropriate. Monotherapy with an aminoglycoside was considered an inappropriate therapy. The 28-day mortality rate was used as an endpoint and it was defined as death occurring within 28 days after bacteraemia onset. For patients who were discharged before the 28-day limit, the status was determined by review of outpatient records or by contacting the patient. No patient was lost to follow-up. Recurrent bacteraemia was defined as the redetection of *A. baumannii* 14 days after the index day.

### Species identification and clonal study

Bacterial isolates were phenotypically identified as members of the Abc using the Vitek 2 system (bioMérieux, Marcy l’Étoile, France). A multiplex-polymerase chain reaction (PCR) method was used to identify *A. baumannii* to the species level^27^. The clonal relationship of clinical isolates was determined by PFGE as previously described^28^. Isolates were considered to be different pulsotypes if they had more than three DNA fragment differences and a similarity of <80% in dendrogram analysis.

### Antimicrobial susceptibility testing

The MICs of antimicrobial agents were determined by agar dilution according to the guidelines of the Clinical and Laboratory Standards Institute (CLSI)^29^. Antimicrobial susceptibility was interpreted according to the standards of the CLSI^30^. MDR was defined as resistance to ≥3 antimicrobial classes: aminoglycosides, antipseudomonal carbapenems, fluoroquinolones, antipseudomonal cephalosporins and β-lactam/β-lactamase inhibitor combinations. The MIC_90_ indicated the concentration of each antimicrobial agent required to inhibit 90% of the strains^29^.

### Biofilm formation and measurement

Biofilm formation experiments were conducted using a microtitre plate assay as previously described^31^ with slight modifications. *A. baumannii* strains were cultured in 5 mL of Luria-Bertani (LB) broth supplemented with 1% D-glucose (LBglu) at 37 °C for 1 day. Thereafter, the cultures were diluted in LBglu to 0.03 at an OD_570_ and 200-μL aliquots were added to each well of a 96-well polystyrene tissue culture plate. The plates were subsequently incubated at 37 °C with shaking (180 rpm) for 48 hours. The suspensions were removed and the wells were washed with phosphate buffered saline (PBS). Thereafter, 200 μL of 0.1% crystal violet in H_2_O was added to stain the cells. The plates were incubated for 20 minutes with gentle agitation, thoroughly washed with PBS and then the stained biofilms were solubilized with 200 μL of 95% ethanol for 10 minutes with gentle agitation. The amount of biofilm formed was quantified by measuring the OD_570_. The OD_570_ values of un-inoculated wells were used as a negative control. We classified isolates as biofilm-forming if the OD_570_ values were at least twice those of the negative controls^14^. All experiments were performed in triplicate and repeated on three separate occasions. When an isolate was positive for biofilm formation on at least two occasions, the isolate was considered to be biofilm-positive.

### Biofilm susceptibility testing

The antimicrobial susceptibility of *A. baumannii* biofilm cells was determined using the MBEC assay as previously described with some modification^32,33^. In brief, the tested isolates were cultivated using a 96-well microtitre plate (with a sterile peg lid) containing fresh Mueller-Hinton broth (MHB) for 16 hours at 37 °C to allow for biofilm formation. Thereafter, the biofilms were treated with serial dilutions of imipenem and meropenem for 20 hours at 37 °C. After incubation, the peg lids were rinsed three times in PBS and placed into antibiotic-free MHB in a flat-bottom microtitre plate. To transfer the biofilms from the pegs to the wells, each plate was centrifuged at 800 × *g* for 20 min and 20 µL was removed from each well and placed in the corresponding well of a fresh, sterile 96-well microtitre plate containing 180 µL of fresh MHB and incubated overnight to allow for recovery. The minimum antibiotic concentration at which no viable cell counts were recovered (OD_570_ <0.07) was considered to be the MBEC. All the experiments were performed in triplicate and repeated on three separate occasions.

### Whole genome sequencing

Biofilm-associated genes and their promoters in biofilm-forming and non-biofilm-forming strains were identified by whole genome sequencing. Bacterial genomic DNA was purified from a single bacterial colony sub-cultured on Muller-Hinton agar and incubated for 16–24 hours using the fully automated LabTurbo 48 Compact System DNA extraction system. Genomic DNA isolated from a well-characterized *A. baumannii* strain was used to prepare a sequencing library in 90 minutes using Illumina’s Nextera DNA Sample Preparation Kit. For sequencing on the MiSeq instrument, prepared samples were placed in the reagent cartridge and loaded on the instrument along with the flow cell. All subsequent steps were performed on the instrument, including cluster generation, single or paired-end sequencing and primary data analysis. For *de novo* sequencing, the *A. baumannii* library prepared using Nextera sample preparation reagents was sequenced using a 2 × 150 read length on the MiSeq. Sequence read data were analyzed and assembled using a pipeline developed specifically for bacterial sequencing. The National Center for Biotechnology Information nucleotide database was searched for *A. baumannii*. Sequences were filtered by position to keep only the genomic regions corresponding to biofilm-associated genes, including *BfmS*, *AbaI, Bap1*, *Bap2*, *PgaABCD* and *CsuAB/ABCDE*. These regions were then organized as a sequence list in the Workbench and converted to a reference track. Post-adaptor-clipped and quality-trimmed reads per sample were then mapped in pairs against this reference track under default parameters. The number of mapped reads and the type of variants per gene falling within each biofilm-associated gene were then enumerated. In addition, the promoter sequences of these genes were also analyzed.

### Multilocus sequence typing (MLST)

MLST scheme of selected isolated were tested as previous described^34^. This scheme involves PCR amplification and sequencing of seven housekeeping genes (*gltA*, *gyrB*, *gdhB*, *recA*, *cpn60*, *gpi* and *rpoD*).

### Quantification of expression of biofilm-associated genes

A qRT-PCR assay was conducted for detection and quantification of mRNA of biofilm-associated genes. Total RNA was isolated using RNAprotect bacterial reagent and an RNeasy Mini kit (Qiagen, Valencia, CA, USA) according to the manufacturer’s instructions. Genomic DNA contamination was eliminated by RNase-free DNase I treatment. RNA was reverse transcribed using random hexamer primers (MBI Fermentas, Vilnius, Lithuania) with Moloney Murine Leukemia Virus reverse transcriptase (Epicenter, Madison, WI, USA). PCR of total RNA without reverse transcription was used to examine the DNA contamination of RNA samples. Real-time PCR was carried out in final reaction volumes of 20 μL with 10 μL of Fast SYBR® Green Master Mix (2×) (Thermo Fisher Scientific Taiwan Co., Ltd. Taipei, Taiwan), 1 μL of each primer set (2 μM), 1 μL of template DNA and 3 μL of ddH_2_O. Thermal cycling was performed on the 7500 Fast (7500 Fast Real-Time PCR System) using the cycling conditions: pre-denaturation at 95 °C for 20s followed by 50 cycles of denaturation at 95 °C for 3s, annealing and extension at 60 °C for 30s. The amplification program was then followed by a melting cycle of 95 °C for 15s, 60 °C for 1 min, 95 °C for 15s and 60 °C for 15s. The qPCR amplification of the targeted DNA was monitored by the increase in the fluorescence in real time. The positive amplification of 16S rRNA gene was determined when the PCR cycle crossed the threshold cycle (*C*_*T*_) value and confirmed by the DNA melt curve analysis. All experiments were performed in duplicate.

### Construction of carbapenem-resistant transformants of *A. baumannii* and Acinetobacter-derived cephalosporinase transformant of *A. baumannii*

To elucidate the impact of carbapenem resistance on biofilm formation, the vectors containing different carbapenemase genes with or without the upstream insertion sequence as a strong promoter were transformed into a biofilm-forming *A. baumannii* reference strain Ab15151 as previously described^35^ and the biofilm-forming capabilities of carbapenem-resistant transformants and Ab15151 with empty vectors were measured. Transformation of the vectors containing ADC genes with the upstream insertion sequence was also performed and the biofilm-forming capabilities of ADC transformant and Ab15151 with empty vectors were compared.

### Statistical analysis

The PASW statistical package for Windows (version 20; SPSS, Chicago, IL, USA) was used for all data analyses. The χ^2^ test with Yates correction or Fisher’s exact test was used to compare categorical variables. Continuous variables were analyzed using the two-sample *t*-test. The time to mortality, defined as the interval between bacteraemia onset and death, was analyzed using the Kaplan–Meier survival analysis and the log-rank test was used to compare univariate survival distribution between different groups of patients. Univariate analyses were performed separately for each risk factor to ascertain the OR and 95% CI. All biologically plausible variables with *p* values less than 0.10 in the univariate analysis were considered for inclusion in the logistic regression model in the multivariate analysis. A backward selection process was utilized. A *p* value less than 0.05 was considered statistically significant.

### Data availability

The datasets generated during and/or analysed during the current study are available from the corresponding author on reasonable request.

## Electronic supplementary material

Supplementary information
