# Supplementary material for: Biofilm formation is not associated with worse outcome in Acinetobacter baumannii bacteraemic pneumonia
Source: Sci Rep. 2018 May 8;8:7289. doi: 10.1038/s41598-018-25661-9 (PMC5940913; doi:10.1038/s41598-018-25661-9)

**Title: Biofilm formation is not associated with worse outcome in *Acinetobacter baumannii* bacteraemic pneumonia**

Yung-Chih Wang,[1,2] Tzu-Wen Huang,[3,4] Ya-Sung Yang,[1] Shu-Chen Kuo,[5] Chung-Ting Chen,[6] Chang-Pan Liu,[7,8] Yuag-Meng Liu,[9] Te-Li Chen,[10,11] Feng-Yee Chang,[1] Shih-Hsiung Wu,[12] Chorng-Kuang How,[6,13] Yi-Tzu Lee.[6,13,*]

**Supplementary information**

**Supplementary Table S1. Use of antimicrobials and the 28-day mortality rates in patients with bacteraemic pneumonia caused by non-biofilm-forming *Acinetobacter baumannii.***

| Main agents used | Patients, no. | APACHE II score, Median (interquartile range) | Patients, no. (%) | | | |
|---|---|---|---|---|---|---|
| | | | Appropriate antimicrobial therapy | Combination antimicrobial therapy | 28-Day non-survivors | 28-Day survivors |
| Anti-pseudomonal penicillins[a] | 24 | 25.46 (19.25–30) | 11 (45.8) | 4 (16.7) | 14 (58.3) | 10 (41.7) |
| Anti-pseudomonal cephalosporins[b] | 29 | 26.62 (21-33) | 4 (13.8) | 9 (31) | 15 (51.7) | 14 (48.3) |
| Anti-pseudomonal fluoroquinolones[c] | 8 | 29.25 (16.5-39.75) | 1 (12.5) | 3 (37.5) | 4 (50) | 4 (50) |
| Anti-pseudomonal carbapenems[d] | 65 | 26.63 (21.5-32) | 19 (29.2) | 14 (21.5) | 37 (56.9) | 28 (43.1) |
| Ampicillin/sulbactam or sulbactam | 19 | 28.16 (22-39) | 6 (31.6) | 10 (52.6) | 11 (57.9) | 8 (42.1) |
| Non-antipseudomonal β-lactamases[e] | 20 | 29.2 (19-38.75) | 8 (40) | 1 (5) | 11 (55) | 9 (45) |

* Data are the median (interquartile range) for continuous variables and number of cases (%) for categorical variables. APACHE II = Acute Physiologic and Chronic Health Evaluation II.

[a]piperacillin, piperacillin/tazobactam, and ticarcillin/clavulanate.

[b]cefoperazone, ceftazidime, cefepime, and cefpirome.

[c]ciprofloxacin and levofloxacin.

[d]imipenem and meropenem.

[e]penicillin, amoxicillin/clavulanate, cefazolin, cefuroxime, cefotaxime, cefmetazole, and flomoxef.

**Supplementary Table S2. The minimum inhibitory concentration (MIC) and minimum biofilm eradication concentration (MBEC) of imipenem and meropenem against 10 *Acinetobacter baumannii* isolates.**

| Drugs | | Imipenem | | Meropenem | |
|---|---|---|---|---|---|
| Strains | Pulsotype | MIC (mg/L) | MBEC (mg/L) | MIC (mg/L) | MBEC (mg/L) |
| 13 | P4 | 64 | >2048 | 128 | >2048 |
| 41 | P5 | 4 | >2048 | 2 | >2048 |
| 93 | P11 | 8 | >2048 | 16 | >2048 |
| 99 | P14 | 64 | >2048 | 64 | >2048 |
| 102 | P15 | 16 | >2048 | 32 | >2048 |
| 137 | P31 | 0.25 | >1024 | 0.25 | >1024 |
| 146 | P36 | 0.5 | >1024 | 0.25 | >1024 |
| 156 | P43 | 64 | >2048 | 0.25 | >1024 |
| 177 | P60 | 1 | 1024 | 4 | >2048 |
| 190 | P68 | 4 | >2048 | 32 | >2048 |

* Values represent the mode of at least 3 biological replicates.

**Supplementary Table S3. Biofilm-associated genes of selected *Acinetobacter baumannii* isolates.**

| | | | | | | | *CsuAB/ABCDE* | | | | | |
|---|---|---|---|---|---|---|---|---|---|---|---|---|
| Isolates | Pulsotype | *BfmS* | *AbaI* | *Bap1* | *Bap2* | *pgaABCD* | *CsuA* | *CsuB* | *CsuC* | *CsuD* | *CsuE* | *CsuA/B* |
| Biofilm-forming isolates (n=9) | | | | | | | | | | | | |
| 32 | P4 | + | + | + | + | + | + | + | + | + | + | + |
| 102 | P15 | + | + | + | + | + | + | + | + | + | + | + |
| 145 | P35 | + | + | + | + | + | + | + | + | + | + | + |
| 154 | P41 | + | + | + | + | + | + | + | + | + | + | + |
| 156 | P43 | + | + | - | - | + | + | + | + | + | + | + |
| 160 | P46 | + | + | - | - | + | + | + | + | + | + | + |
| 171 | P56 | + | + | + | + | + | + | + | + | + | + | + |
| 177 | P60 | + | + | + | + | + | + | + | + | + | + | + |
| 181 | P61 | + | + | + | + | + | + | + | + | + | + | + |
| Total, no. (%) | | 9 (100%) | 9 (100%) | 7(77.8%) | 7(77.8%) | 9 (100%) | 9 (100%) | 9 (100%) | 9 (100%) | 9 (100%) | 9 (100%) | 9 (100%) |
| Non-biofilm-forming isolates (n=30) | | | | | | | | | | | | |
| 3 | P1 | + | + | + | + | + | + | + | + | + | + | + |
| 6 | P2 | + | + | + | + | + | + | + | + | + | + | + |
| 21 | P4 | + | + | + | + | + | + | + | + | + | + | + |
| 49 | P5 | + | + | + | + | + | + | + | + | + | + | + |
| 74 | P6 | + | + | + | + | + | + | + | + | + | + | + |
| 83 | P7 | + | + | + | + | + | + | + | + | + | + | + |
| 90 | P9 | + | + | + | + | + | + | + | + | + | + | + |
| 94 | P12 | + | + | + | + | + | + | + | + | + | + | + |
| 100 | P14 | + | + | + | + | + | - | - | - | - | - | - |
| 103 | P15 | + | + | + | + | + | + | + | + | + | + | + |
| 105 | P16 | + | + | + | + | + | + | + | + | + | + | + |
| 107 | P17 | + | + | + | + | + | + | + | + | + | + | + |

| | | | | | | | | | | | | |
|---|---|---|---|---|---|---|---|---|---|---|---|---|
| 114 | P20 | + | + | + | + | + | + | + | + | + | + | + |
| 124 | P23 | + | + | + | + | + | + | + | + | + | + | + |
| 129 | P25 | + | + | - | - | + | - | - | + | + | + | + |
| 131 | P26 | + | + | + | + | + | + | + | + | + | + | + |
| 133 | P28 | + | + | - | + | + | + | + | + | + | + | + |
| 135 | P29 | + | + | + | + | + | - | - | - | - | - | - |
| 140 | P33 | + | + | + | + | + | + | + | + | + | + | + |
| 142 | P34 | + | + | + | + | + | + | + | + | + | + | + |
| 144 | P35 | + | + | + | + | + | + | + | + | + | + | + |
| 152 | P40 | + | + | + | + | + | + | + | + | + | + | + |
| 158 | P45 | + | + | + | + | + | + | + | + | + | + | + |
| 161 | P47 | + | + | + | + | + | + | + | + | + | + | + |
| 164 | P50 | + | + | + | + | + | + | + | + | + | + | + |
| 166 | P52 | + | + | + | + | + | + | + | + | + | + | + |
| 170 | P55 | + | + | + | + | + | + | + | + | + | + | + |
| 182 | P62 | + | + | + | + | + | + | - | + | + | + | + |
| 186 | P64 | + | + | + | + | + | - | - | - | - | - | - |
| 187 | P65 | + | + | + | + | + | + | + | + | + | + | + |
| Total, no. (%) | | 30 (100%) | 30 (100%) | 28 (93.3%) | 29 (96.7%) | 30 (100%) | 26 (86.7) | 26 (86.7) | 27 (90%) | 27 (90%) | 27 (90%) | 27 (90%) |

**Supplementary Fig S1. Pulsed-field gel electrophoresis of 190 isolates of *Acinetobacter baumannii* showed two major clones, pulsotype P4 and P5. BF = biofilm-formation, NBF = non-biofilm-formation.**

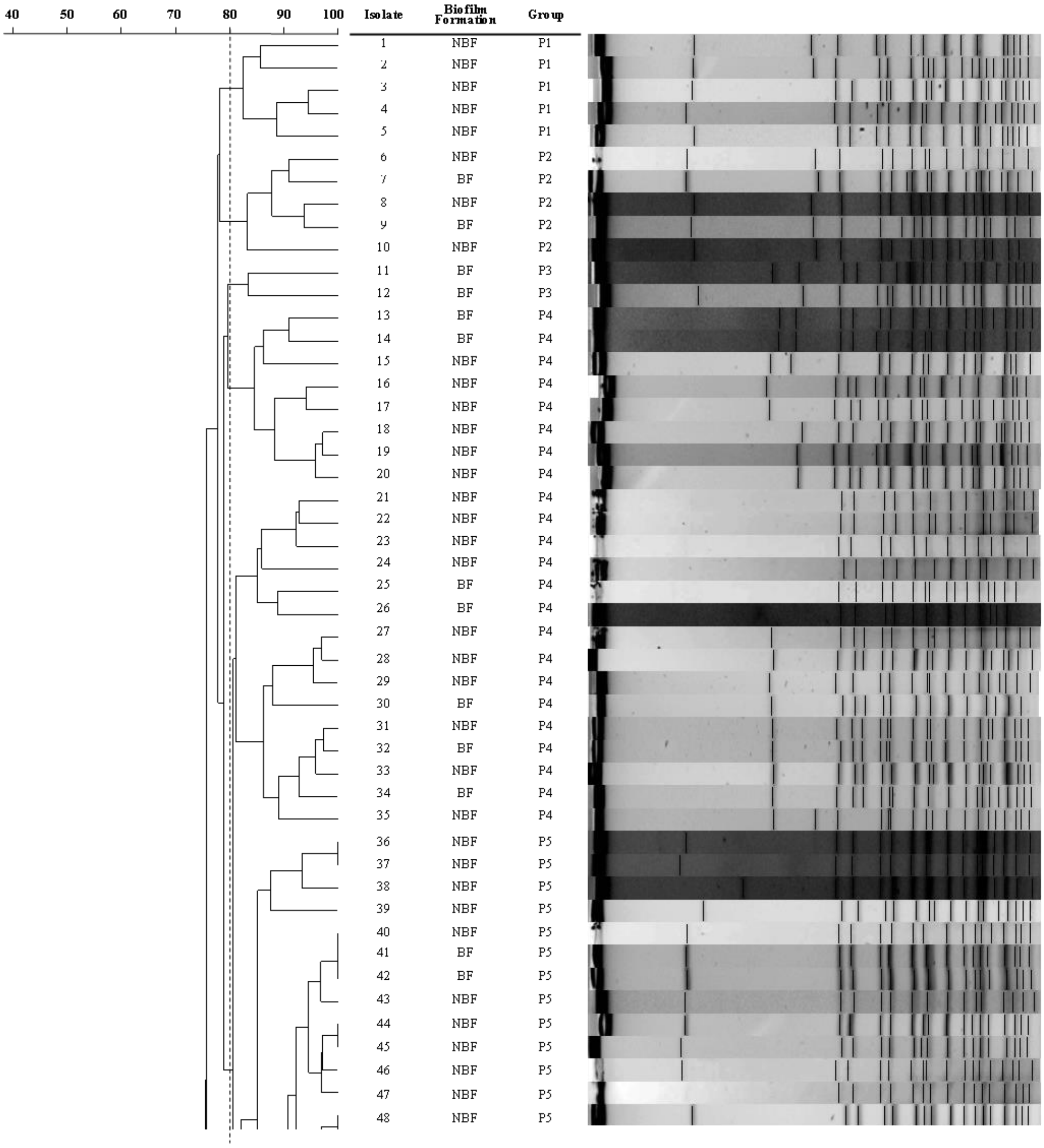

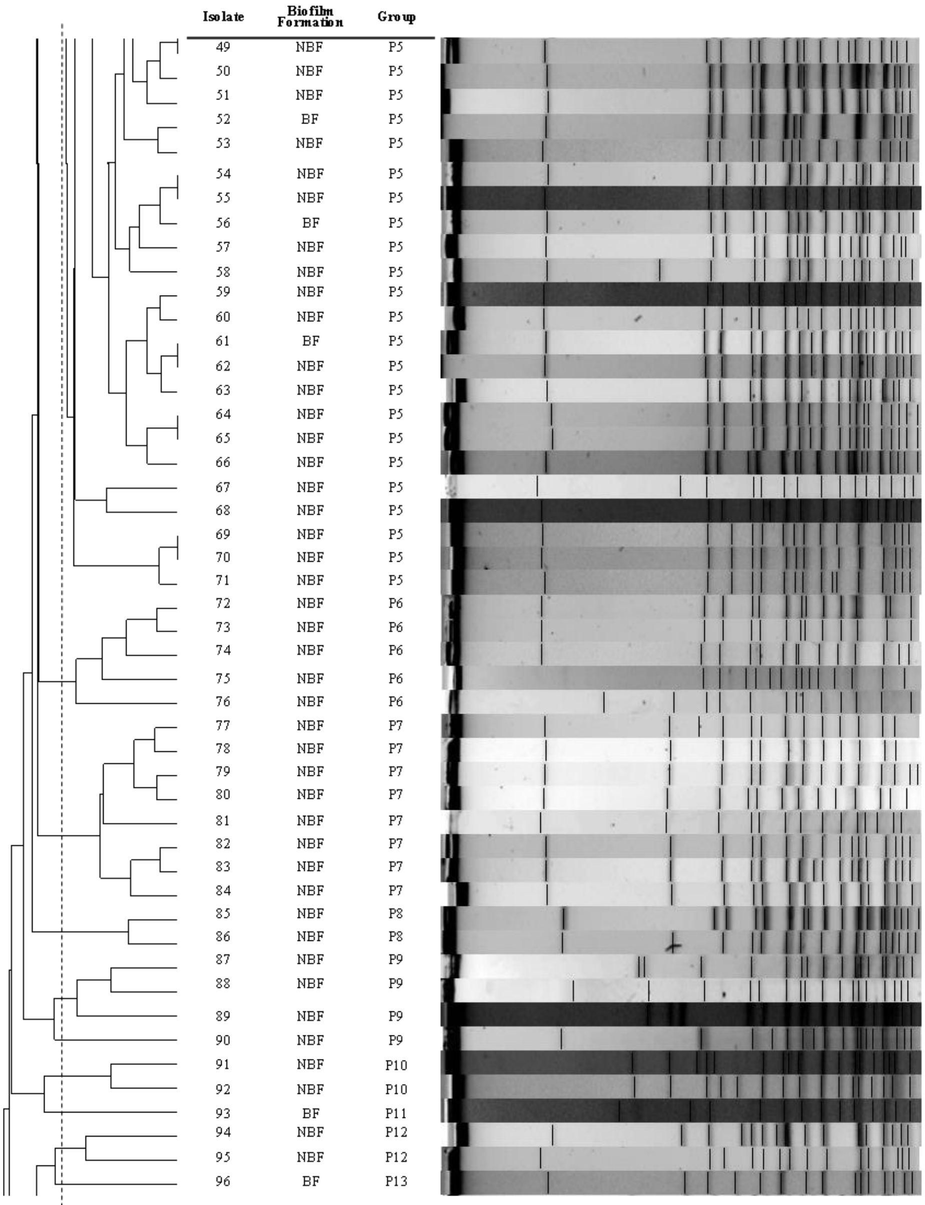
Isolate
Biofilm Formation
Group
49 NBF P5
50 NBF P5
51 NBF P5
52 BF P5
53 NBF P5
54 NBF P5
55 NBF P5
56 BF P5
57 NBF P5
58 NBF P5
59 NBF P5
60 NBF P5
61 BF P5
62 NBF P5
63 NBF P5
64 NBF P5
65 NBF P5
66 NBF P5
67 NBF P5
68 NBF P5
69 NBF P5
70 NBF P5
71 NBF P5
72 NBF P6
73 NBF P6
74 NBF P6
75 NBF P6
76 NBF P6
77 NBF P7
78 NBF P7
79 NBF P7
80 NBF P7
81 NBF P7
82 NBF P7
83 NBF P7
84 NBF P7
85 NBF P8
86 NBF P8
87 NBF P9
88 NBF P9
89 NBF P9
90 NBF P9
91 NBF P10
92 NBF P10
93 BF P11
94 NBF P12
95 NBF P12
96 BF P13

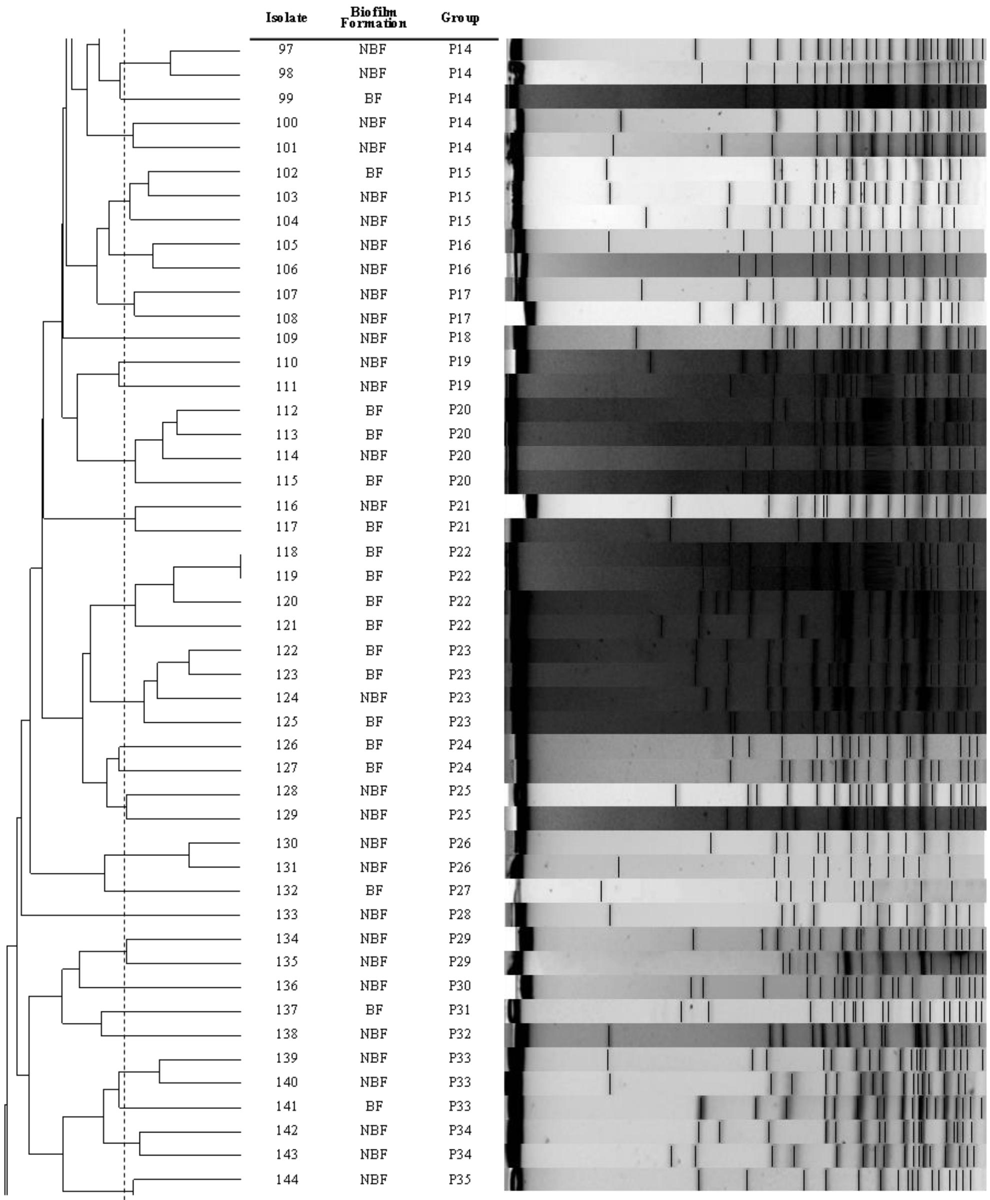
Isolate
Biofilm Formation
Group
97 NBF P14
98 NBF P14
99 BF P14
100 NBF P14
101 NBF P14
102 BF P15
103 NBF P15
104 NBF P15
105 NBF P16
106 NBF P16
107 NBF P17
108 NBF P17
109 NBF P18
110 NBF P19
111 NBF P19
112 BF P20
113 BF P20
114 NBF P20
115 BF P20
116 NBF P21
117 BF P21
118 BF P22
119 BF P22
120 BF P22
121 BF P22
122 BF P23
123 BF P23
124 NBF P23
125 BF P23
126 BF P24
127 BF P24
128 NBF P25
129 NBF P25
130 NBF P26
131 NBF P26
132 BF P27
133 NBF P28
134 NBF P29
135 NBF P29
136 NBF P30
137 BF P31
138 NBF P32
139 NBF P33
140 NBF P33
141 BF P33
142 NBF P34
143 NBF P34
144 NBF P35

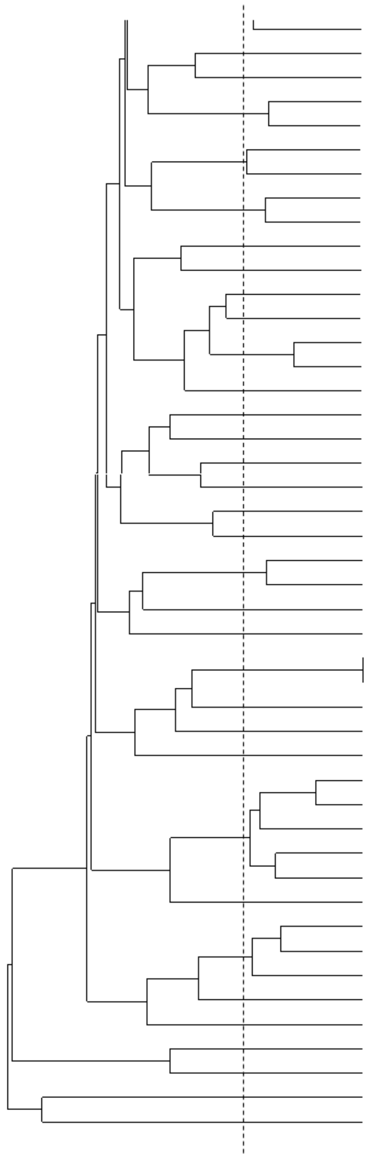

| Isolate | Biofilm Formation | Group |
|---|---|---|
| 145 | BF | P35 |
| 146 | BF | P36 |
| 147 | BF | P37 |
| 148 | BF | P38 |
| 149 | NBF | P38 |
| 150 | NBF | P39 |
| 151 | NBF | P39 |
| 152 | NBF | P40 |
| 153 | NBF | P40 |
| 154 | BF | P41 |
| 155 | NBF | P42 |
| 156 | BF | P43 |
| 157 | BF | P44 |
| 158 | NBF | P45 |
| 159 | BF | P45 |
| 160 | BF | P46 |
| 161 | NBF | P47 |
| 162 | NBF | P48 |
| 163 | BF | P49 |
| 164 | NBF | P50 |
| 165 | BF | P51 |
| 166 | NBF | P52 |
| 167 | BF | P53 |
| 168 | BF | P53 |
| 169 | BF | P54 |
| 170 | NBF | P55 |
| 171 | BF | P56 |
| 172 | BF | P56 |
| 173 | BF | P57 |
| 174 | BF | P58 |
| 175 | BF | P59 |
| 176 | BF | P60 |
| 177 | BF | P60 |
| 178 | BF | P60 |
| 179 | BF | P60 |
| 180 | BF | P60 |
| 181 | BF | P61 |
| 182 | NBF | P62 |
| 183 | BF | P62 |
| 184 | BF | P62 |
| 185 | NBF | P63 |
| 186 | NBF | P64 |
| 187 | NBF | P65 |
| 188 | NBF | P66 |
| 189 | NBF | P67 |
| 190 | BF | P68 |

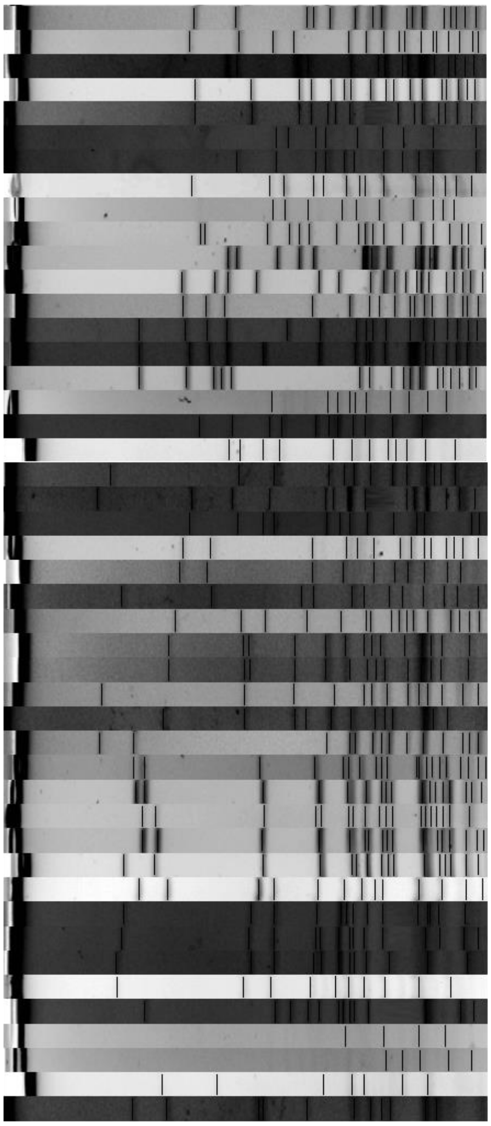

**Supplementary Fig S2. Carbapenem-resistant transformants harboring various carbapenemase genes produced less biofilm at 48 h than the *Acinetobacter baumannii* ATCC 15151(Ab15151) carrying an empty vector.**

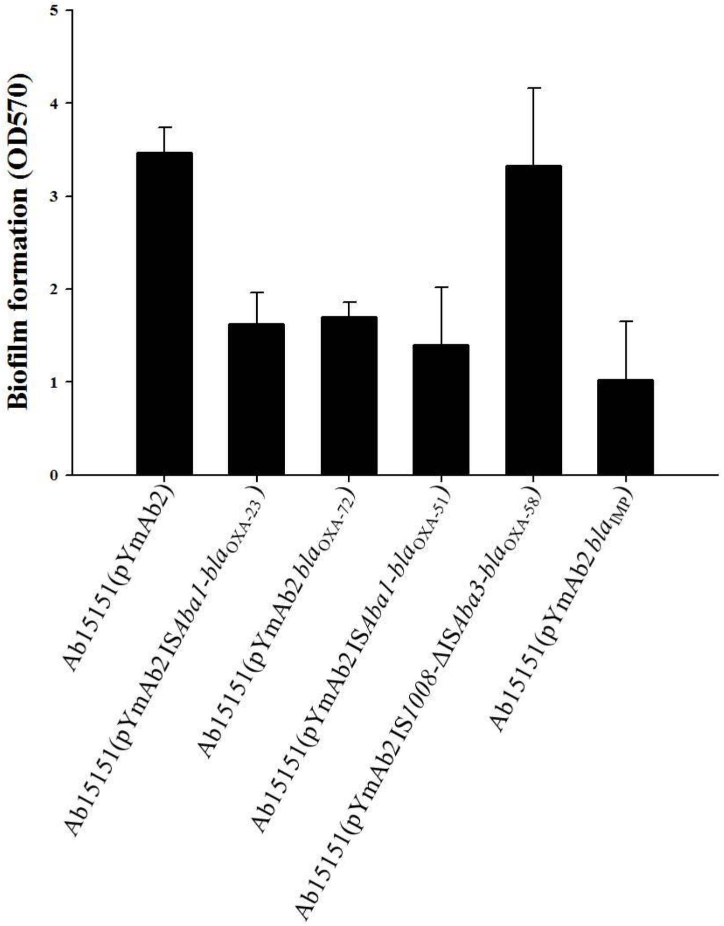

**Supplementary Fig S3. Quantitative reverse transcription polymerase chain reaction (qRT-PCR) showed that the expression levels of (a)-(b) *bap1* [fold change 1.99 and 1.73, respectively], (c)-(d) *abaI* [fold change 2.01 and 1.52, respectively], and (e)-(f) *csu A/B* [fold change 8.2 and 5.42, respectively] were higher in the biofilm-forming isolates (isolates 32 and isolates 42) than in the matched non-biofilm-forming isolates (isolates 81 and isolates 84).**

**(a) *bap1***

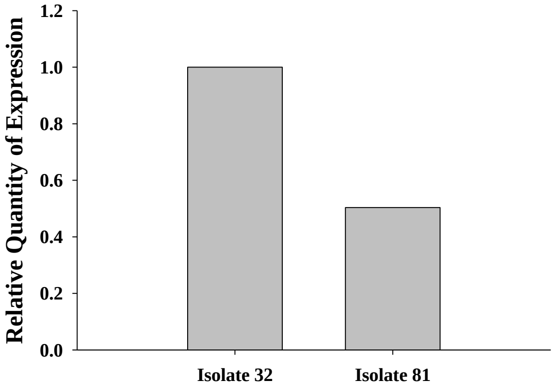


**(b) *bap1***

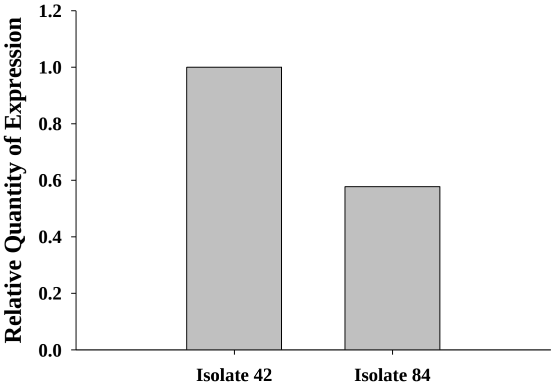


**(c) *abaI***

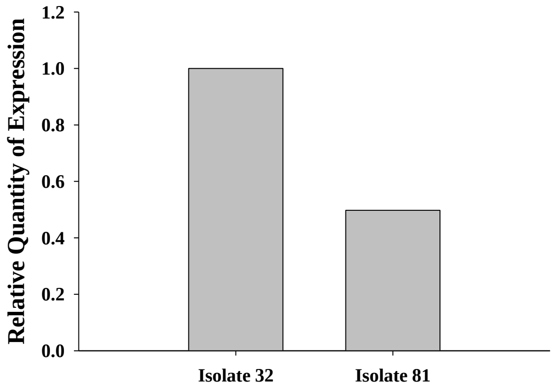


**(d) *abaI***

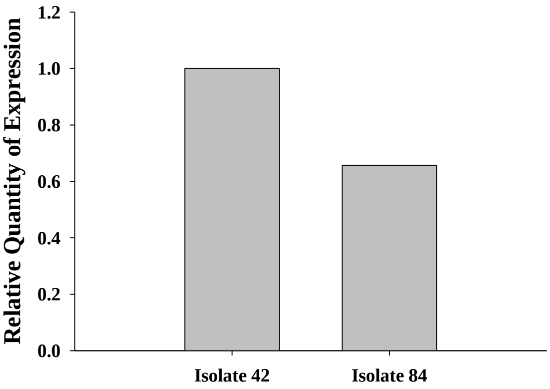


**(e) *csuA/B***

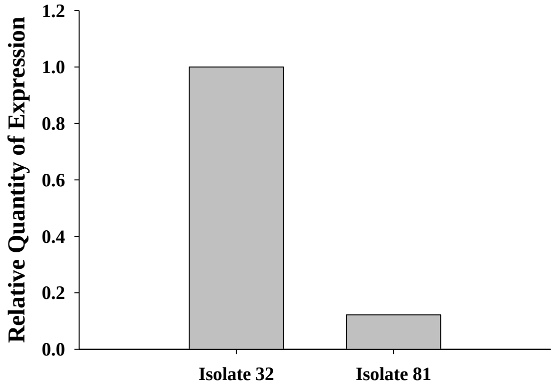


**(f) *csuA/B***

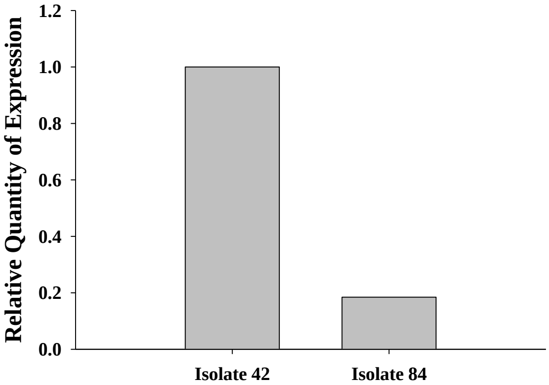

Supplement: Supplementary file 1 — Supplementary information [file 41598_2018_25661_MOESM1_ESM.pdf]
